# Amanitins: The Most Poisonous Molecules of the Fungal World

**DOI:** 10.3390/molecules28155932

**Published:** 2023-08-07

**Authors:** János Vetter

**Affiliations:** Department of Botany, University of Veterinary Medicine, Pf. 2., 1400 Budapest, Hungary; vetter.janos@univet.hu

**Keywords:** octapeptide amanitins, affected mushrooms, chemistry, occurrence, contents of toxins, course of poisoning, therapy

## Abstract

Among the toxic metabolites of the fungal world, those that, due to their strong biological effect, can seriously (even fatally) damage the life processes of humans (and certain groups of animals) stand out. Amatoxin-containing mushrooms and the poisonings caused by them stand out from the higher fungi, the mushrooms. There are already historical data and records about such poisonings, but scientific research on the responsible molecules began in the middle of the last century. The goals of this review work are as follows: presentation of the cosmopolitan mushroom species that produce amanitins (which are known from certain genera of four mushroom families), an overview of the chemical structure and specific properties of amanitins, a summary of the analytical methods applicable to them, a presentation of the “medical history” of poisonings, and a summary of the therapeutic methods used so far. The main responsible molecules (the amanitins) are bicyclic octapeptides, whose structure is characterized by an outer loop and an inner loop (bridge). It follows from the unusual properties of amanitins, especially their extreme stability (against heat, the acidic pH of the medium, and their resistance to human, and animal, digestive enzymes), that they are absorbed almost without hindrance and quickly transported to our vital organs. Adding to the problems is that accidental consumption causes no noticeable symptoms for a few hours (or even 24–36 h) after consumption, but the toxins already damage the metabolism of the target organs and the synthesis of nucleic acid and proteins. The biochemical catastrophe of the cells causes irreversible structural changes, which lead to necrotic damage (in the liver and kidneys) and death. The scientific topicality of the review is due to the recent publication of new data on the probable antidote molecule (ICR: indocyanine green) against amanitins. Further research can provide a new foundation for the therapeutic treatment of poisonings, and the toxicological situation, which currently still poses a deadly threat, could even be tamed into a controllable problem. We also draw attention to the review conclusions, as well as the mycological and social tasks related to amanitin poisonings (prevention of poisonings).

## 1. Introduction

In the world of fungi, we can find many types of toxin-producing species and their toxic metabolic products. All these causes many problems (mycotoxicoses) for human and veterinary medicine, microbiology, and other scientific disciplines. Fungal toxins of peptide character produced by mushrooms (mainly *Amanita* species) play a prominent role [1,2,3]. The question of the documented occurrence of cyclic peptides in mushrooms, especially the mycological background of toxin-containing genera (species), needs to be reviewed—or even criticized [4]. This prominent role is explained not only by some of the characteristics of these molecules (extreme stability against temperature, pH, digestive enzymes; special cyclic peptide structure, etc.), but also by their very strong biological effects. The biological effects extend to humans and specific groups of animals (mammals). For the detection of toxic compounds and the reliable (and fast) measurement of their quantity, new analytical possibilities are being developed and applied, and this area must also be reviewed [5,6]. In the event of an uncertain diagnosis, other methods also come to the fore, for example, the identification of fungal pieces that have entered the body using molecular methods [7]. Human amatoxin poisonings are also among the most difficult to treat since antidote molecules against the active substances have not been found to date. During the preparation of this review, a very important study was published in mid-May 2023 [8]. This publication reports on the promising protective effects of a molecule (previously used for radically different medical diagnostic purposes), indocyanine green, against the toxic effects of amatoxins.

The essence of the treatment protocol for amatoxin poisoning used so far is to reduce the symptoms and damage, protect the liver and kidneys, and even perform a liver transplant [9,10,11]. Although the mortality rate of the poisonings is decreasing, it is still high, even though the mechanism of action of the responsible amatoxins (inhibition of the RNA polymerase II enzyme) is finally clear. Even in this situation, prevention is particularly important, which includes the reliable identification of these mushrooms in time and the avoidance (prohibition) of their consumption, since the best therapeutic method is if the poisoning does not even occur.

Our review aimed at a broad presentation of amanitin-producing mushrooms and the molecules in question using new scientific knowledge, relying on the chemical, medicinal, and veterinary medicinal aspects of our previously published articles on the subject [12,13].

## 2. Brief Historical Background

R. Hallermayer (Ph.D. thesis, 1940) was the first to describe the successful crystallization of the active ingredient “amanitin” from the poisonous mushroom *Amanita phalloides*, all of which took place in the laboratory led by H. Wieland at the University of Munich. The chemical nature of the mushroom’s toxins was established at the beginning of the twentieth century. W.W. Ford (Baltimore, MD, USA) began to study it in the case of a similar poisonous mushroom, the *Amanita virosa* (destroying angel) species. Ford named this thermostable molecule amanitatoxin; 0.4 mg of the partially purified American preparation caused death in a guinea pig when administered subcutaneously [14]. Professor Wieland and the author of the Ph.D. dissertation reported on the results of their work in an important publication [15].

The historical roots of death cap poisoning date back much further. We cannot know exactly when the first serious or fatal poisoning occurred, but there are interesting historical facts and records. The first known case may have been the death of the Roman emperor Claudius (AD 53), when the symptoms following the consumption of food containing mushrooms were presumably caused by the death cap. It is strange that at that time the emperor’s favorite dish, the Caesar’s mushroom (*Amanita caesarea*), was served, and perhaps the extract of the death cap mushroom could have been added to it. An example in Hungarian history is the German-Roman Emperor, Charles VI, who was on a hunting trip in Hungary (1740) and, after dinner, fell ill and died within a few days, probably because of death cap poisoning.

## 3. Amanitin-Producing Mushrooms

Amanitin-containing mushrooms can be found in one genus in each of the four basidiomycetous families (Amanitaceae, Agaricaceae, Bolbitiaceae, and Hymenogastraceae). The poisonings that occurred after the consumption of mushrooms understandably focused attention on species with a higher toxin content. These taxa are more toxic and are very common, as we saw in the historical background, and the species *A. phalloides*, *A. virosa*, and *A. verna* appeared first on the list. Not only did the more frequent occurrence of these mushrooms “help” to clarify their role, but the social attitude towards mushroom consumption (mycophilic or mycophobic ones) of the given ethnic group also had an impact. Obviously, the extraction and detection of toxins, as well as the reliable measurement of their concentration, require an appropriate methodological background and preparation. Let us not forget that the health preparedness, experience, and toxicological background of the given area (country) also have an impact on the situation. Some of the mushrooms in the first-mentioned Amanitaceae family are widespread on all continents. The *Amanita* genus may include 900–1000 species [16], but, to date, only approx. half of them have been noted. The large number of probably existing but undescribed species may also mean that among the species to be known later, they may also contain amatoxin, so the number of species can be expected to increase. The genus *Amanita* is divided into seven sections by mycology, partly on morphological and partly molecular grounds, of which the Phalloideae section contains the toxin-producing species [16]. A good example of the expansion of toxin species is the publication from South Korea [10], in which the species *A. virgineoides* and *A. cheelii* (former: *A. punctata*) were added to the list of toxin-containing species in connection with specific poisonings.

The cosmopolitan *Lepiota* genus of the Agaricaceae family includes 300–400 species, which can be classified into six sections (Echinatae, Fuscoviolaceae, Lepiota, Lilaceae, Ovisporae, and Stenospore) [17]. These species, which live on nutrient-rich soil, are not among those tempting for consumption, but unfortunately, they can also be mistaken for mushrooms such as *Macrolepiota procera* or *Tricholoma terreum* [17]. Already at the end of the last century, studies drew attention to the fact that some species contain amatoxins. Garcia et al., in their work presenting amatoxin-containing mushrooms [18], list 9 *Amanita*, 21 *Lepiota*, and 8 *Galerina* species. However, according to the latest analytical work [17], no toxin was detected in six mushrooms previously considered to contain amatoxins, so their role is questionable [18]. In the case of the *Lepiota brunneoincarnata* and *L. venenata* species, Chinese studies from 2020 [19] showed a direct cause-and-effect relationship in cases of poisoning with acute liver damage caused by these species, so these taxa are among the significant amatoxin carriers.

Summarizing the above, it can be concluded that changes to the list of amatoxin-containing species can be expected because of new research and even documented cases of poisoning around the world: new species may be added, or it may even happen that one species is removed from the list [17]. Based on the literature [2,3,10,16,20,21,22,23,24], the currently “valid” list of toxin-containing species is compiled in Table 1, taking the official Latin names and descriptors of the mushrooms from the Index Fungorum database [23].

**Figure 1:**
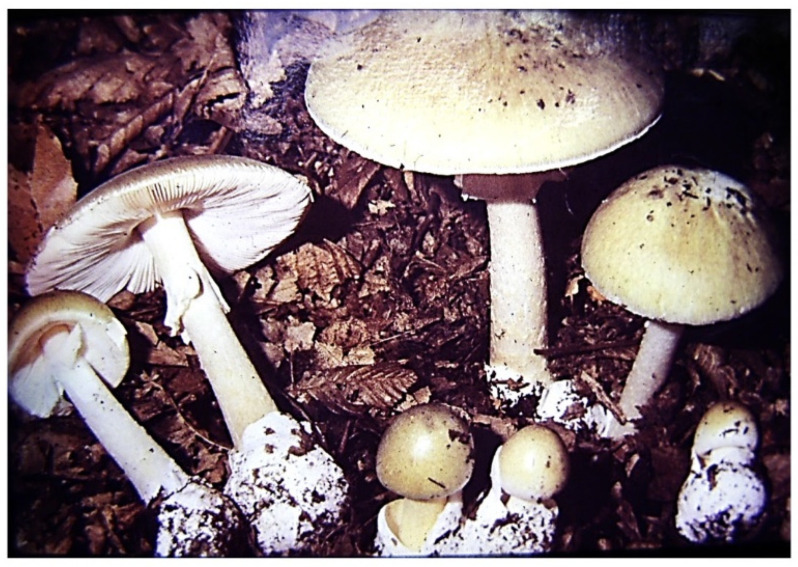
*Amanita phalloides* (death cap) mushroom.

**Figure 2:**
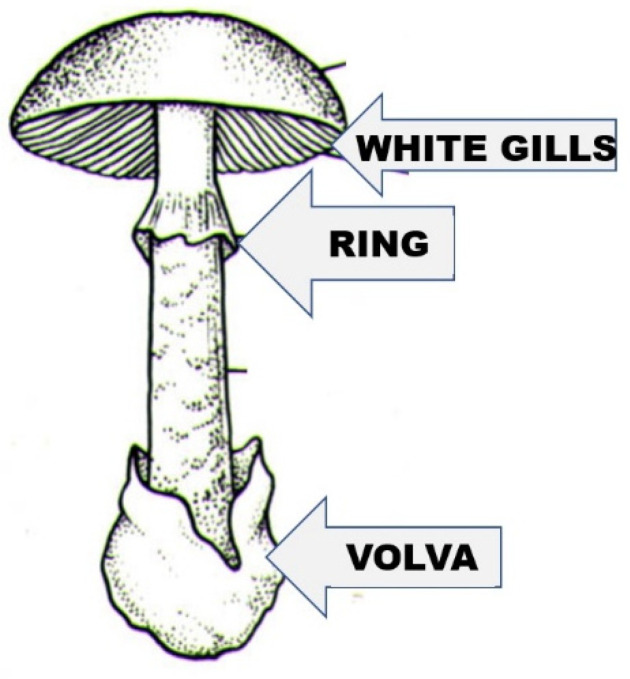
The characteristic morphological properties of the species (white gills, ring, and volva), the combined presence of which clearly indicates the death cap mushroom.

The purpose of this work is not—nor is it possible—to provide a detailed description and presentation of amatoxin-containing mushrooms, but rather to make one exception. This can be none other than the most cosmopolitan species containing the most amatoxins, as well as the most frequently reported poisoning cases: *A. phalloides* (death cap). It lives in a symbiotic relationship (ectomycorrhiza), mainly with deciduous trees (in Middle Europe with oak species, beech, and hornbeam), and less often with conifer species. The northern limit of its distribution is approx. the same as that of the oak species (61 degrees north latitude). It is also widespread on other continents (North America, North Africa, and North Asia), but occurs in Central and South America, South Africa, and has also been transferred to Australia and New Zealand. The cap of the fruiting body (Figure 1) is 5–15 (on average 10) cm wide, convex, and later flattens. Its color varies greatly; often it has a greenish hue, but whitish, yellowish, greenish brown, and even other shades of brown can occur. Due to the great variability of color, it is not advisable to consider it a defining property when identifying the mushroom. The cap peel is radially fibrous. smooth, shiny, and slightly sticky in wet weather. The gills of the mushroom are white; their color remains white even in old age, and they stand freely and densely. Its stipe is between 7 and 20 cm long, cylindrical, and its surface often has olive-green, brownish belt-like, “snakeskin-patterned” zones. Its ring (annulus) is white and membranous, and the tuberous base of the stipe has a white volva. The flesh is white and juicy; the smell is sweet (reminiscent of raw potatoes or fruits); and the taste is pleasant (but should not be tasted). We would like to clarify the sure recognition and identification of the mushroom with Figure 2, where its three characteristic features are highlighted: the joint presence of the always white gills, of the ring, and of the volva indicates that the mushroom is the death cap.

## 4. Chemistry

Although the concept of amatoxin includes three large groups of molecules (amanitins, phallotoxins, and virotoxins) [12], only amanitins are the focus of this work. The explanation for this is that the biological effects of phallotoxins and their intensity are significantly lower than those of amanitins, and virotoxins practically do not cause harmful biological effects when they enter the human body.

**Figure 3:**
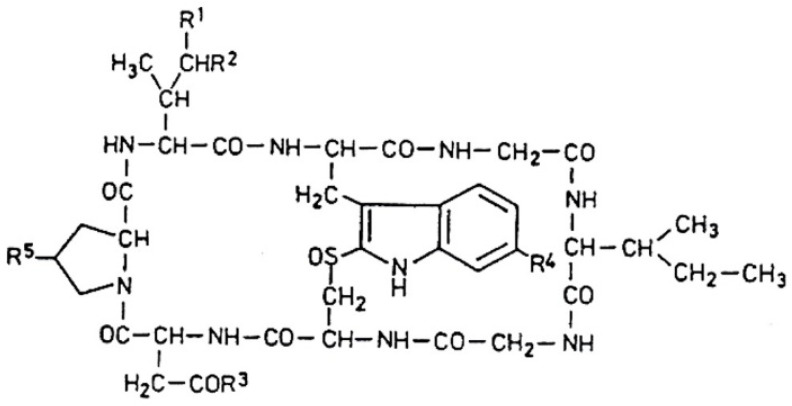
Basic structure of amanitin (based on [12]); see also the content of Table 2.

Amanitins are bicyclic octapeptides, the basic structure of which can be seen in Figure 3. The different substituents are in five positions (**R^1^–R^5^**) of the molecule, which are presented in Table 2. There are a total of nine derivatives: α-, β-, γ-, and ε-amanitins; amanin; amanin amide; amanullin; amanullic acid; and proamanullin. The molecular weight of the derivatives—with minor differences—is around 900 Da. Two groups of the presented derivatives can be distinguished: neutral amatoxins, such as α- and γ-amanitins, amanin amide, amanullin, and proamanullin; and acidic molecules, such as β- and ε-amanitins, amanin, and amanullic acid [18]. The structures of the derivatives differ based on the number of OH groups and the replacement of carboxyl-amide groups in the R_3_-position. Three amino acids (amino acid derivatives) with an unusual structure occur: 4-hydroxyproline, dihydroxy-isoleucine, and 6-hydroxy-triptophan [21]. The amino acid sequence of α-amanitin is Ile-Trp-Gly-Ile-Gly-Cys-Asn-Pro, and that for β-amanitin is Ile-Trp-Gly-Ile-Gly-Cys-Asp-Pro.

The most important toxicological studies were carried out with α- and β-amanitin. The bicyclic nature of the structure (Figure 3) means that an outer and an inner loop are formed in the structure consisting of eight amino acids. The outer loop of this structure is created by the peptide bonds between the C- and N-termini of the amino acids. The inner loop is formed by the “triptathionine” bond between 6-hydroxytryptophan and cysteine. It is an unusual, intramolecular sulfur-containing “bridge” [26]. The question of solubility is very important in relation to the effect, efficiency, and subsequent fate of the molecules. In general, the presence of hydroxyl groups on the side chains of molecules causes solubility. Generally, amatoxins dissolve well in water, methanol, and other organic solvents (including ethanol, DMSO/dimethylsulfoxide), but they are insoluble in certain organic solvents (ether, chloroform, etc.).

The presented aminitin group is characterized by some unusual properties that fundamentally influence the behavior of molecules entering animals and humans, i.e., the possibility of developing toxic effects. One of these is that the stability and resistance of these molecules to heat treatments (cooking, roasting, etc.) are very high.

The stability of the toxin α-amanitin was investigated at different temperatures (at 25 °C, at 4 °C, at −20 °C, and in conditions of boiling water) [27]. The initial amount of toxin was 100%, and the remaining toxin quantity was measured after the different treatments. The remaining toxin levels were 86% and 96% after 6 months at room temperature, in water and methanol, respectively. The storage at 4 °C after 6 months produced a higher quantity of undamaged toxin (91% and 97%, respectively, in water and methanol).

If the storage was at a temperature of −20 °C, the amount of remaining toxin was 76% and 97% in water and methanol, respectively. Only 5% of the toxin remains after 6 h of cooking. According to the authors, the chemical structure of the molecule changes because of long cooking, but this does not necessarily mean a reduction in toxicity.

In recent investigations by Sharma and coworkers [28], death cap samples were treated with various heat and technical conditions (frying, grilling, boiling, baking). The duration of these treatments was 30 min, but the temperatures used were not published. The amatoxin concentrations of baked mushrooms were higher than those of grilled or oil-fried samples. The amatoxin level in boiled samples was the lowest, i.e., this is where most amatoxin loss occurred.

The likely explanation is that amanitins are water-soluble, and boiling for half an hour extracted and transported the toxin from the mushroom into the cooking water (a similar phenomenon can also occur when preparing mushroom food, but in this case, the aqueous medium is also part of the food). The consumer later eats most of it, which then enters the body. The other treatments at higher temperatures (frying in oil, grilling, or frying) reduced the amanitin concentrations by 20–30%. All this also means that heat treatment procedures at higher temperatures for a longer period can reduce the amount of the toxin (in contrast to our previous view, which considered the heat stability of amatoxins almost unlimited).

The molecules remain stable even at an acidic pH (during technical operations in the kitchen). There is no change in the structure of the molecules as a result of human digestive enzymes, i.e., the molecules are not even partially broken down in the digestive tract [12,13]. The bicyclic peptide structure may be behind all these effects.

## 5. Determination of Amanitins

The analysis of amanitins (generally amatoxins) was not an easy task in connection with poisonings caused by toxins and toxin-containing mushrooms in general. There should be suitable, sensitive, and specific methods that can be used to identify toxins and quickly measure their concentration in mushrooms (possibly in the available mushroom food residue) and, in the case of poisoning, from several biological samples (stomach content, blood/plasma, urine).

**Figure 4:**
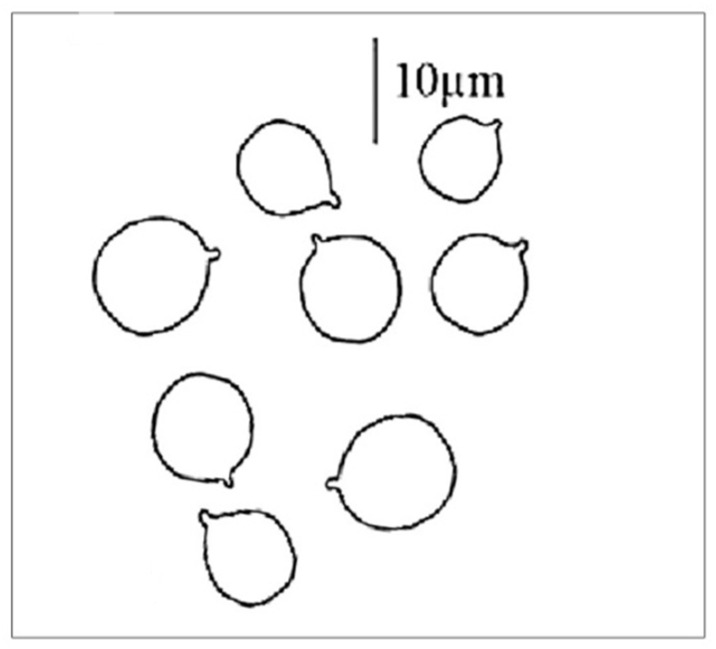
Spores of death cap (*Amanita phalloides*). The line represents 10 µm.

Classic methods are also available to identify the mushroom, thus confirming or even ruling out the type of suspected poisoning. Thus, it is also possible to identify the mushroom (food residue) based on the spore morphology and size using the traditional microscopic method. A great advantage of this method is that the suspected species can be confirmed or even excluded within a few minutes. Its disadvantage is of an objective nature, namely that the spore of *A. phalloides* does not show characteristic, easily distinguishable morphological features, as shown in Figure 4. The spores are sub globose to broadly elliptical, with dimensions of 7.7–10.1 µm × 6.7–8.5 µm [29]; the spore powder is white, the spore wall is smooth, and it does not contain surface structures (spore ornamentation is lacking). However, there are opinions that draw attention to the problems of the microscopic identification of spores [30]. Analysis of materials containing *Amanita phalloides* spores documented that they have high similarity to oil drops and/or other cell structures present in biological samples. According to opinions [30], the absence of death cap spores in the tested samples does not rule out the possibility of this poisoning, and some kind of instrumental analysis is recommended.

**Meixner test**. For the detection of amanitin (amatoxin)-containing mushrooms, the so-called Meixner’s test was used earlier, but it is still sometimes mentioned, referred to, and even used in some places (mainly in mycology). The Meixner test [31,32] (also referred to as the Wieland or Wieland/Meixner test) is based on the color reaction of cyclopeptides and lignin in the paper (wood) in the presence of concentrated hydrochloric acid. Wieland previously used paper with a high lignin content; after adding the mushroom extract (or juice), the added HCl caused a blue spot on the paper. Meixner experienced a similar coloration on newsprint, although the resulting coloration can also be greenish blue (this was considered a positive result). The reaction of the coniferyl aldehyde with the lignin molecules in the paper and the indole ring in the toxin was written as an explanation for the color [31]. Several problems arose during the application of the test; sometimes a color developed even without the addition of the mushroom extract. Further doubts arose when it became clear that, in many cases, false-positive reactions were observed. Certain molecules can cause a color change, for example, pyrroles (red), indole derivatives (cherry red), resorcinol (blue), and phenol derivatives (blue). In addition, the quality of the paper used (lignin content) made the reproducibility expected from the method very difficult. The test’s simplicity, speed, and lack of equipment made it quickly popular, especially among mushroom pickers. However, our opinion today is that its use for the reliable testing of poisonous mushrooms and/or the diagnosis of specific cases of poisoning is outdated and should be discarded [18,31,33].

### 5.1. Chromatographic Methods

The development of chromatographic methods in the 1950s and 1960s made it possible to separate and isolate a wide variety of molecules, mainly of plant origin, and then to determine them semi-quantitatively and later quantitatively. Today, one-third of the publications containing amatoxin determinations provide data on mushrooms, and two-thirds provide biological samples from mushroom poisonings [21]. Two-thirds of the applied analytical methods are of the chromatographic type, and one-third are not based on chromatographic methods. All Amanita toxins were detected by paper chromatography, but such methods had low resolution, and the accuracy of the quantitative determinations was also inadequate. In the 1960s, the thin layer chromatography (TLC) method was used, where the resolving power was better, and it was mainly used in the analysis of toxins in mushrooms (mushroom extracts). It also turned out that TLC methods are not or are barely suitable for examining biological samples of poisoned people. Professor Wieland worked on a silica gel plate, first using a solvent mixture of butanol–ethyl acetate–water (14:12:4) and then chloroform-methanol-water (65:5:4) for separation [34]. A combination of chromatographic methods was used to analyze the methanolic extract of the mushroom, which was first purified on a Sephadex column and then further purified on a thin-layer plate.

Stijve et al. [21] developed a fast and sensitive HPTLC for the determination of α- and β-amanitin in the methanolic extract of *A. phalloides*. The next stage of chromatographic toxin analysis is the development of the HPLC method, to which different detectors can be connected. It was first reported by Bentler and Marderosian [35]. The common feature of these methods is that the stationary phase of the column retains lipophilic amatoxins and separates different molecules (charged/uncharged; polar/apolar compounds).

Thin-layer chromatography (TLC) methods were mainly applied to mushroom extracts, but for biological (medicinal) samples (blood, serum, plasma, stomach contents, and urine), they were not very suitable because they contained significantly smaller amounts of toxins and different disturbing substances were presented.

Later, Wieland [34] successfully used cellulose powder as a medium in a method that can already be classified as liquid chromatography. Barbosa and coworkers [21] in their review work provide a detailed table of the methods used and published in the literature, including conditions, materials, etc., of the studies. The common feature of these methods is that toxin separation was carried out by reversed-phase liquid chromatography under gradient conditions. Each method has different characteristic properties (composition of the mobile phase, flow rate, temperature, gradient elution, etc.). The task and responsibility of the team performing the work are to develop, check, and validate the conditions specifically applied to the given object. Mass spectrometry linked to liquid chromatography has been the most frequently used and successful combination of methods in recent years. It is characterized by separation efficiency and good selectivity, but it also provides structural information (data), molecular weight, and other data.

The development of the methods used for the determination of toxins and the development and application of new methodological possibilities are often based on the weaknesses of the already used methods; they do not always have beneficial properties or meet the needs of therapeutic practice. In the case of amatoxin poisoning, the concentration of the toxin in urine is higher than in other body fluids. If urine is not available because, for example, renal function is decreased or acute renal failure exists, the examination of blood plasma comes to the fore. However, this requires a much more sensitive method.

This was reported by Bambauer and his group [36], who developed a liquid chromatography (LC) high-resolution tandem mass spectrometry (HRMS/MS) method. Sample preparation involved protein precipitation, aqueous liquid–liquid extraction, and solid-phase extraction. The chromatographic separation was carried out by reversed-phase chromatography, and an orbitrap-based MS was used for toxin identification and quantification. The limit of identification was 20 pg/mL (picogram/mL). α-Amanitin could be identified in all blood plasma samples at concentrations between 37 and 2890 pg/mL. This method is very sensitive; it can be crucial because hospitalization is often late after consumption and only very low toxin content can be found.

### 5.2. Nonchromatographic Methods

The ancestor of these is the Meixner (Meixner/Wieland) test, but due to its many problems (see earlier), colorimetric (spectrophotometric) methods were not developed. Several immunoassay methods were used for amatoxin analysis: the radioimmunoassay (RIA), the enzyme-linked immunosorbent assay (ELISA), and, more recently, the lateral flow assay (LFA). This method group started with RIA (47 years ago), where the detection limit of amatoxins was ~0.5 ng/mL. This method requires the production of antibodies, and the procedures are laborious and time-consuming. The ELISA technique was the first attempt to determine the amatoxin components of urine [37]. The ELISA method is simple and sensitive, but it is time-consuming and involves specific chemicals and multiple rounds of incubation and washing. A monoclonal antibody production study was published recently [38]. This antibody binds specifically to amatoxin molecules, and a method was created that determines the amatoxins by an indirect, competitive immunoassay. The method determines α-, β-, and γ-amanitins with detection limits of 4.55 ng/mL, 4.90 ng/mL, and 4.45 ng/mL, respectively, for the three amanitins. The latest methodological combination is lateral flow immunoassay (LFIA) technology. Due to its speed, simplicity, and relatively low cost, its application is spreading rapidly. The applicability of the method is the rapid detection of amatoxins in urine samples, with a detection limit of 10 ng/mL for α- and γ-amanitin and 100 ng/mL for β-amanitin [39]. The great advantage of the method is that it does not require pretreatments, and no special equipment is needed to evaluate the results. Also, 110 wild mushrooms were tested using the same method, and α- and γ-amanitins were detected in all six species known to be toxic [40].

The pretreatments required for amatoxin analyses could be presented in a separate review, which is not possible in the present framework. However, the proper preparation of mushrooms or biomedical objects, their conditions, materials, etc., are important, as they essentially determine the applicability and accuracy of subsequent analytical methods. Detailed material can be found in the new review by Barbosa et al. [21].

## 6. Contents and Distribution of Toxins in Mushrooms

The classical data of Seeger and Stijve [41] refer to fruiting bodies of *A. verna* collected from 11 habitats in Switzerland. These data contained an average of 1.55 mg/g DM for α-amanitin, 1.29 mg/g DM for β-amanitin, 0.37 mg/g DM for γ-amanitin, and 3.2 mg/g DM for the total toxin content. *A. phalloides* samples were also collected, and their toxin contents (5.2–7.4 mg/g DM) were significantly higher than the 3.4–4.5 mg amanitin/g DM values of the *A. verna* samples.

It is worth comparing the previous results (data) with much later analyses of Turkish samples [42]. The authors of the latter work fractionated the fruit bodies in terms of morphology: they divided the pileus, gills, stipe, and volva and analyzed the whole fruit body. Compared to the data of the classical analysis, its approximate value was measured threefold in whole fruiting bodies. According to data from Turkey [42], α-amanitin is the toxin present in the largest proportion (66–75%), the rate of β-amanitin is 24–32%, and γ-amanitin has the smallest proportion (3–11%) in the whole fruiting body and in all morphological fractions. The reported toxin ratios are approximately the same as those found in classic studies, although more β-amanitin was measured then (see previous paragraph). Regarding the distribution of toxins in morphological parts, the order is as follows: gills (13.38 mg/g DM) > pileus (10.16 mg/g DM) > whole fruiting body (9.96 mg/g DM) > stipe (9.99 mg/g DM) > volva (2.85 mg/g DM).

The inhomogeneous nature of toxin distribution in the individual parts of the fruiting body was already indicated in previous studies [43], since the death cap was similarly fractionated, showing that the cap, gills, ring, and stipe are characterized by high concentrations of amanitin, while the volva and the lower part of the stipe are characterized by low concentrations. The analyzed samples came from three different habitats with different geological and soil conditions, and the toxin contents of the three sample groups differed from each other.

The species *A. fuliginea*—originally described in Japan—is a highly poisonous species that causes most of the fatal poisonings in southern China today. The amanitin contents in samples from six Chinese habitats [23] are 4.22 ± 1.82 mg/g DM, 1.27 ± 0.37 mg/g DM, and 5.50 ± 2.10 mg/g DM for α- amanitin, β- amanitin, and total amanitins, respectively. α-Amanitin accounts for 76% of the average total content and β-amanitin accounts for 24%, so the ratio of the two amanitins is 3:1. According to the development of α-amanitin levels in the fruiting body fractions, the order is as follows: gills (10.29 mg/g DM) > stipe (6.89 mg/g DM) > pileus (6.84 mg/g DM) > annulus (2.32 mg/g DM) > volva (1.38 mg/g DM) > spores (0.13 mg/g DM).

Regarding the changes in toxin concentrations during development, the total toxin concentration increased gradually during the early developmental stages; the maximum value (11.6 mg/g DM) was reached when the mushroom was in vigorous growth and then decreased when the fruiting body was mature.

Twenty samples of ten Amanita species were analyzed for α- amanitin, β- amanitin, and total amanitin contents [2]. Most of the samples came from Chinese habitats, but some originated from European and American habitats, and there were some that were examined after 15–20 years of storage (storage conditions are unknown). If we aggregate according to the species (based on the total amanitin content), they are located on a wide scale: between 1.37 and 10.51 mg/g DM content (from *A. phalloides* until *A. rimosa*). The α-amanitin accounts for 53–81% of the total content; β-amanitin content is between 19 and 49%. The species *A. virosa* contains only α-amanitin; β-amanitin was not found in any of its samples.

Fruiting bodies of *A. phalloides var. alba* collected in Turkey [44] were separated into caps, gills, stipe, and volva; spores and the entire fruiting body were also analyzed. The order of total amanitin (α + β + γ) concentrations in the above fractions is as follows: gills (4.76 mg/g DM) > cap (4.42 mg/g DM) > whole fruiting body (4.16 mg/g DM) > stipe (2.73 mg/g DM) > spores (1.38 mg/g DM) > volva (0.99 mg/g DM). In the case of the whole fruiting body, 51%, 41%, and 8% characterized the proportions of α-, β-, and γ-amanitins, respectively. Very similar amanitin ratios characterize the other fruiting body fractions, i.e., the distribution of amanitin types can be said to be almost constant.

Although the literature data refer to several species from different places of production and the determination methods were not the same, they showed several matching trends. Among amanitins, the order of concentration α > β > γ almost always follows; among the morphological parts, the cap, the gills, and the ring contain the most toxin amounts, and the stipe, volva, and spores have the lowest toxin levels. Many studies indicate that environmental factors (geological, soil, climatic, and ecological conditions) obviously influence toxin amounts and that growth can be measured during the development of the mushroom, which already decreases in the mature fruiting body phase.

## 7. Amatoxin Poisoning

Amatoxin poisoning begins at the moment of consumption. The consumption must be considered an accident, because neither the mushroom picker nor the user recognized the deadly poisonous mushroom.

The symptoms of poisoning can be divided into four main stages [45]:

1. The relatively long latency period (Lag stage) is on average 8–10 h, and in a few cases it is longer (24–36 h). During this time, there are no signs of poisoning, and, in fact, proportionate to the length of the period, the consumer thinks less and less about the mushrooms eaten earlier (even 1–2 days before).
2. The second stage is the gastrointestinal phase (GI stage). Main characteristic GI symptoms: intensive, repeated vomiting, abdominal pain, watery diarrhea, leading to dehydration and hypovolemic shock; hypoglycemia can develop. The duration of the GI phase is 1–2 days. A problematic (and dangerous) point is that if the diagnosis of this situation is missed, the patient may be misdiagnosed (the problem is gastroenteritis) and discharged [45].
3. The symptoms of the GI phase slowly subside, but the toxins attack the liver and kidneys more and more strongly, and the activities of liver enzymes (GOT, GPT, and LDH) increase rapidly. The coagulation of blood is disturbed, which may induce internal bleeding.
4. In the last stage (multiorgan failure stage), many deteriorating metabolic parameters indicate that the effects of toxins are spreading there is an increase in liver transaminase, LDH, bilirubin levels, disorders in coagulation, bleeding, metabolic acidosis, hypoglycemia, and encephalopathy. Physiological parameters indicate increasingly extensive liver (and kidney) damage, which may be partially irreversible. Death can occur 6–10 days after consumption.

Previous studies [12] clearly clarified that fatty degeneration, acute liver dystrophy, and centrilobular necrosis were present in the livers of poisoned people. When analytical possibilities became available for rapid examination of the biological samples of the patients, it turned out that significant changes could be measured in the development of toxin concentrations. The concentration of α-amanitin in the patients’ plasma varied between 8 → 190 ng/mL and 21 → 102 ng/mL in the concentrations of β-amanitin, 38 h after consuming the mushroom. Magnitude changes are observed in urine and fecal toxin concentrations when toxin excretion increases.

In veterinary practice, amatoxin poisoning of varying severity has been reported in different animal species [13]. There is more uncertainty regarding the occurrence of animal poisonings, since even in the case of our pets, there is little chance that the owner will notice the moment when the nimble animal has just bitten and swallowed a piece of mushroom. In the case of farm animals, it is unlikely that the farmer will notice the case or provide any information to the veterinarian in the event of symptoms appearing later. Several publications report on amatoxin poisoning in animals (dogs: [46,47]; cats: [48]; cattle: [49,50]).

According to the LD_50_ values of amatoxins [13], the human value of 0.1 mg/kg body weight is the same as the sensitivity of rabbits and guinea pigs (0.1–0.2 and 0.05–0.1, respectively). LD_50_ values of amatoxins (in mg/kg body weight) for dogs (depending on the breed): 0.1; pigs: 0.1–0.2; cats: 0.5; mice: 0.4–0.8; rats: 2.8–3.5; amphibians (frogs): between 2 and 5; while mollusks have an LD_50_ of 20 mg/kg body weight. It is also clear that the LD_50_ value of a snail is about 200 times the human value. It follows from this that a snail consuming a poisonous species does not indicate the edibility of the mushroom, but its LD_50_ value is high.

What is experienced in cases of poisoning in dogs is largely the same as human experience; the course is faster, and post-mortem examinations showed deaths in the liver and kidney tubules, gastritis, and hemorrhages under the serous membrane [13]. Beagle dogs were poisoned by administering lyophilized *A. phalloides* samples back in the 1980s [51]. After the gastrointestinal symptoms, the increased enzyme activities, bilirubin level, and prothrombin time indicated that after 48 h, very severe liver damage developed, which led to the deaths of four of the eight animals between the 35th and 54th hours. In the case of cats, the livers and kidneys of animals showed serious, lethal changes, and, unlike dogs, inflamed, dead sections were also visible in some parts of the gastrointestinal tract. The available data for cattle is very incomplete, but it is interesting that, for example, in California, the number of amanitin poisonings of cattle during the 11-year period was 2.5% of the registered poisonings. After a latency of eight to twelve hours, strong gastrointestinal symptoms begin; after a few hours of improvement, liver and kidney damage (liver enzyme activities, increased clotting time) and hypoglycemia appear. During the period of acute liver damage, effective intervention was no longer possible [49,50].

There is only one literary reference to amanitin poisoning in horses [52], where the poisoning and subsequent death of the horse are explained by a previously existing brain tumor (meningioangiomatosis) because the horse changed its general nutritional habits and consumed mushrooms.

**Toxokinetics.** The absorption of amatoxins in the gastrointestinal tract is very fast, and the toxins appear in the urine 90–120 min after mushroom consumption. The toxins do not bind to albumins, but their quantity quickly decreases in the blood, and they are transported to the liver and kidneys. According to dog experiments, it is known that the half-life of amatoxins is between 27 and 50 min, and their detectability in the blood is possible for 4–6 h [18,53].

The absorbed amatoxins are primarily transported to the liver via the OATP1B3 and NTCP transporter systems [53]. Meanwhile, most of the amatoxins are excreted within the first 72 h of poisoning. Relatively few toxins enter the bile, which is then reabsorbed in the intestinal system. The toxin concentrations that can be measured in kidney cells are 6–90 times higher than in the liver [53].

**Mechanism of action**. It is basic information that the amount of RNA continuously decreases during the first 24 h of poisoning because the activity of RNA polymerase type II is reduced; thus, mRNA and protein synthesis are also reduced. In a classic experiment [12], it was proven that amanitin given in an amount of 10 ng/mL reduces the activity of the RNAP II (EC 2.7.7.6) enzyme by 60–70%. Following the connection between the toxin and the enzyme, a non-covalent nuclear inhibition is created. In another classic experiment [12], the tritium-labeled amatoxin molecule was fixed by its carboxyl group to the enzyme. An amatoxin-binding protein has been detected in calf thymus nuclei. It is an important fact that the RNA polymerase enzymes of eukaryotes essentially differ in their sensitivity to amatoxin molecules. RNAP II is the most susceptible of the three (I, II, and III) enzymes. RNAP II, originating from mammalian cells, fish, and insects, is highly sensitive to α-amanitin; the sensitivity of non-mammalian polymerase enzymes is slower. It is interesting that mushrooms in general, including the *Amanita* species, are resistant to their own inhibitors.

According to Wielend’s classical experiments [12], the binding sites of amatoxin molecules are on the concave side of the 24-membered macrocycle. The affected molecular parts are the γ-hydroxyl of -γ-δ-dihydroxyleucine-3, the oxygen 08 of hydroxyprolin-2, and two carbonyl oxygens and two methyl carbons of isoleucine-6.

What are the consequences of RNAP II inhibition? Amatoxins induce necrosis in the livers of all mammals. Dog liver cell cultures were tested under different concentrations of α-amanitin. After six hours of treatment, concentrations of 5–40 µM are effective, and after 24–48 h, concentrations of 1–5 µM are also effective: cell viability, total protein, and urea levels decrease, and LDH activity increases. According to electron microscopy (EM) studies, even the six-hour treatment with 1 µM caused a variety of ultrastructural changes: there are pale, foam-like areas in the cytoplasm, the chromatin content of the cell nuclei is condensed, and depending on time and toxin concentration, an increasing number of necrotic cells are visible. In these cells, the endoplasmic reticulum (ER) breaks down, and the mitochondria swell and become disorganized.

The effect of amanitins is two-stage: first, functional damage (inhibition of protein synthesis) occurs, followed by a chain (series) of cellular damages. In the second stage, necrotic or apoptotic death characterizes the cells. It is possible that the mechanism of action of amatoxins is even more complicated than the above; for example, some of the amatoxins are transformed in the liver into derivatives that increase the number of oxygen radicals. A higher number of free radicals can lead to further damage [13].

## 8. Therapy of Amatoxin Poisonings

The beginnings of amatoxin poisoning therapy can take place in several ways, depending on how the poisoning was discovered. It is possible that a seriously poisonous mushroom was placed on the table before the mushrooms were eaten. Medical treatment for the poisoned person, who is still asymptomatic at this time, must begin immediately. It is possible—and in most cases, this is the case—that the problem begins later with the perception of symptoms in the GI phase. In terms of health intervention, it is essential to determine whether there is any uncertainty about amatoxin-containing mushrooms or whether the situation is clear. If the species of the mushroom is uncertain, the situation must be clarified so that treatment in the right direction can begin as soon as possible.

The main goal of previous therapeutic procedures was to improve the patient’s condition and replace fluid and electrolyte losses. Partial removal of absorbed amatoxins can (and should) begin as soon as possible with activated carbon treatment and various biochemical methods (hemodialysis, hemoperfusion, and plasmapheresis). More recently, an extracorporeal purification system (MARS: Molecular Adsorbent Recirculating System) has been used. The aim of this is the transfer of toxic metabolites from the blood stream into a dialysate compartment by a specific membrane. The general experience is that detoxification outside the body is only effective if one begins using it very early.

Chemotherapy steps, i.e., the use of various substances (natural and synthetic molecules), are another important element of the therapies. The literature (54) discusses and reviews a wide variety of chemotherapy options. In the past (and in some cases even today), this is how high doses of antibiotics (penicillin G), antioxidants (vitamin C, thioctic acid), hormones, and steroids [54] were administered.

Milk thistle (*Silybum marianum)* is a medicinal plant from the Mediterranean region belonging to the Asteraceae family. The active ingredients are flavonoids (flavonoglycans) in the ripe fruits (seeds), the main component of which is **silibinin**. The latter molecule is a mixture of two diastereomers: silybin A and silybin B. The physiological effect of silibinin is complex, as follows:

a. Stabilizes the membranes of liver cells; the entry of toxins into the cells is strongly inhibited.
b. Has a pronounced antioxidant effect; lipid peroxidation is inhibited, and the synthesis of inflammatory substances is reduced.
c. Helps the regeneration of liver cells, accelerates protein synthesis, and helps the regeneration of damaged membranes [55].

Silibinin treatment is recommended within 48 h after mushroom consumption (20–50 mg/kg/day, intravenously), for at least 48–96 h.

In human and veterinary medicine, the use of the plant’s active ingredients is recommended not only in cases of amatoxin poisoning, but also in general for protecting liver function and promoting its regeneration.

The molecules mentioned and used in therapy are not considered antidotes but according to experience, they improve the chances of poisoning, mainly due to their liver protection and/or antioxidant effects.

From the mid-1990s on, liver transplantation became a practical option if the serious condition of the liver justified it. Transplantation is justified if the prothrombin time is very prolonged and conditions of metabolic acidosis, hypoglycemia, and increased serum ammonia exist. The two main options for liver transplantation are orthotopic and partial liver transplantation. Deciding the necessity and nature of liver transplantation is not an easy task, the details of which are far beyond the goals and possibilities of this work.

On the other hand, it is necessary to deal with a question that may bring (or can bring) a fundamental change in the treatment of those poisoned by amatoxin and in the issues of such poisonings in general. In mid-May 2023, a team of mostly Chinese researchers [8] published a voluminous publication. In connection with amatoxin poisoning, researchers report the possibility of using indocyanine green (ICG), a compound previously used for other medicinal purposes, as an antidote for α-amanitin. ICG is a diagnostic reagent that has existed for many decades and is used mainly in ocular angiography and hepatic function assessment. The molecule has no obvious side effects; at a dose of 0.5 mg/kg, the LD_50_ value for mice is 60–80 mg/kg.

The authors of [8] show that the STT3B enzyme (catalytic enzyme for N-glucan biosynthesis) is required for α-amanitin toxicity and the ICG can be used as a specific antidote molecule. They demonstrated [8] that ICG could block amanitin-induced cell death in vitro and in vivo, suggesting that ICG may have practical importance in the possible treatment of amanitin poisoning.

The presented facts [8] so far only indicate the possibility of a new molecule acting as an antidote. Obviously, long research work and a lot of time are still needed so that the reported findings bring new possibilities to the treatment of poisoning.

## 9. Conclusions

Fatal or serious mushroom poisonings are almost always caused by the accidental consumption of amatoxin-containing mushroom genera, especially *Amanita* (*Lepiota*, *Galerina*) species. Among the amatoxins, molecules of the amanitin group, mainly α-, β-, and γ-amanitins, are behind the poisonings. Amanitins are bicyclic peptides consisting of eight amino acids. Their properties include heat stability, stability in acidic conditions, and stability against human (animal) digestive enzymes. Amanitins can be detected in different amounts in different parts of the mushroom fruiting bodies, mostly in the cap and the ring, and the least in the stipe and the spores. At the beginning of the measurement of the amanitins—which are the most important and best studied from an efficacy point of view—paper and then thin-layer chromatography methods were used, and later, HPLC methods with different detections were developed. Today’s methodological palette is very broad, which is also good because not only mushrooms (mushroom dishes) but also biological samples from humans (and animals), including the blood, urine, stomach contents, and faces, can be tested.

In the case of poisoning or a suspicion thereof, the basic task is to clarify the role of the mushroom quickly and clearly, as this determines the direction, methods, etc., of the treatments. It has been known for decades that the main target of amatoxins is the mammalian enzyme RNA polymerase II, whose inhibition causes serious changes in nucleic acid and protein metabolism. These seriously affect the functions of the liver and kidneys; the cells enter a necrotic state, which leads to the death of poisoned people (or animals) in a significant percentage of cases.

The essence of the therapeutic procedures of the last decade—in addition to stabilizing the patient’s condition—is to reduce and remove the amount of absorbed toxins using various methods (activated carbon, biochemical cleaning, filter methods). An important element of the therapy is the use of chemotherapeutic molecules that, partly based on previous experience, can improve the patient’s condition: high-dose antibiotics, antioxidants, and substances that protect the function of the liver (silibinin). The discovery in 2023 places a new antidote molecule (ICG) at the center of the question because it was established that this molecule can successfully stop the α-amanitin reaction chain through enzyme inhibition.

Intensive research is needed in the following areas:

a. Targeted studies to detect additional amatoxin-containing mushrooms, especially in the Amanita genus.
b. Intensive investigation of the ICG molecules and their applicability as an antidote against α-amanitin in human and veterinary medicine.

## Figures and Tables

**Table 1:** The amatoxin-containing mushrooms (based on data from [2,3,10,16,20,21,22,23,24,25]).

Mushrooms	Species (Official Name According to “Index Fungorum”)
1. Genus *Amanita* (fam.: Amanitaceae)	*A. bisporigera* G.F. and Atk. 1906 *A. brunneitoxicaria* Thongbai, Raspé and K.D. Hyde 2017 *A. cheelii* P.M. Kirk 2013 *A. exitialis* Zhu L. Yang and T.H. Li 2001 *A. ocreata* Peck 1909 *A. phalloides* Vail. ex Fr. 1821 *A. suballiacea* (Murill) Murill 1941 *A. subjunquillea* S. Imai 1933 *A. subpallidorosea* Hai J. Li 1935 *A. tenuifolia* (Murill) Murill 1945 *A. verna* Secr. 1833 *A. virgineoides* Bas. 1969 *A. virosa* Bertill. 1866 *A. hygroscopica* Coker, 1917 *A. fuliginea* Hongo, 1953 *A. pallidorosea* P. Zhang and Zhu L. Yang 2010 *A. cokeri* E.-J. Gilbert and Kühner ex E.J. Gilbert 1940 *A. fuligineoides* P. Zhang and Zhu L. Yang 2010 *A. rimosa* P. Zhang and Zhu L. Yang 2010 *A. brunnescens* G.F. Atk. 1918
2. Genus *Lepiota* (fam.: Agaricaceae)	*L. brunneoincarnata* Chodat and C. Martin 1889 *L. subincarnata* J.E. Lange, 1940 *L. venenata* Zhu L. Yang and Z.H. Chen 2018 *L. elaiophylla* Vellinga and Huijsen 1998 *L. spiculata* Pegler, 1983 *L. farinolens* Bon and G. Riousset 1992 *L. bondieri* Bres. 1884
3. Genus *Galerina* (fam.: Hymenogastraceae)	1. *G. marginata* (Batsch) Kühner 1935
4. Genus: *Conocybe* (fam.: Bolbitiaceae)	1. *C. filaris* (Fr.) Kühner 1935

**Table 2:** Chemical structure of the amatoxins (see Figure 3).

Compounds	Substituents				
	R^1^	R^2^	R^3^	R^4^	R^5^
α-Amanitin	CH_2_OH	OH	NH_2_	OH	OH
β-Amanitin	CH_2_OH	OH	OH	OH	OH
γ-Amanitin	CH_3_	OH	NH_2_	OH	OH
ε-Amanitin	CH_3_	OH	OH	OH	OH
Amanin	CH_2_OH	OH	OH	H	OH
Amanin amide	CH_2_OH	OH	NH_2_	H	OH
Amanullin	CH_3_	H	NH_2_	OH	OH
Amanullinic acid	CH_3_	H	OH	OH	OH
Proamanullin	CH_3_	H	NH_2_	OH	H

## Data Availability

Not applicable.
